# Feasibility analysis of treating breast cancer patients with breast-conserving surgery via a periareolar incision combined with non-lipolytic suspension-type mastoscopy

**DOI:** 10.1038/s41598-023-39199-y

**Published:** 2023-07-26

**Authors:** Jiaqi Liu, Guijin He, Yiwen Zhang, Michael Pak-kai Wong, Jun Chu, Linna Kong, Maya Mazuwin Yahya

**Affiliations:** 1 Universiti Sains Malaysia, USM Health Campus, Kubang Kerian, Kota Bharu, Kelantan Malaysia; 2 Department of Second Breast Surgery, Shengjing Hospital of China Medical University, Shenyang, Liao Ning China; 3 Changchun University of Chinese Medicine, Changchun, JiLin China; 4 Department of Surgery, School of Medical Sciences, Health Campus, Universiti Sains Malaysia, 16150 Kubang Kerian, Kelantan Malaysia; 5 Department of Surgery, Hospital Universiti Sains Malaysia, Kubang Kerian, Kelantan Malaysia; 6 Breast and Thyroid Department, Zibo Central Hospital, Zibo, Shandong China; 7 Breast Cancer Awareness And Research Unit (BestARi), Hospital Universiti Sains, Kubang Kerian, Kelantan, Malaysia

**Keywords:** Cancer, Diseases, Oncology

## Abstract

The purpose is to analyze and compare postoperative recovery and complication incidence between a periareolar incision combined with Suspension-type Mastoscopic Axillary Lymph Node Dissection (SMALND) and traditional inflated Mastoscopic Axillary Lymph Node Dissection (MALND). This was a randomized trial conducted from June 1, 2020, to April 30, 2022, in the Department of Second Breast Surgery, Shengjing Hospital of China Medical University, and the Department of Thyroid and Breast Surgery, Zibo Central Hospital, in accordance with the criteria of inclusion and exclusion. Overall, 126 patients diagnosed and treated for early-stage breast cancer were selected to undergo periareolar-incision breast-conserving surgery. Those patients who underwent periareolar-incision surgery combined with SMALND formed the observation group (SMALND Group), while those who underwent periareolar-incision surgery combined with traditional inflation became MALND Group. In the two groups, paired data “t” was used to examine, analyze, and compare the postoperative daily drainage volume and drain removal time, while paired data “χ^2^” was used to examine, analyze, and compare the incidences of postoperative upper limb edema and paresthesia. There were 64 cases in the SMALND Group and 62 cases in the MALND Group. Between the two clusters, no differences were found in age, clinical staging, BMI, and breast cancer classification (*P* > 0.05). The intraoperative surgery time of the SMALND Group was 43.37 ± 6.27 min while that of the MALND Group was longer: 45.72 ± 4.25 min (*P* < 0.05). The intraoperative hemorrhage volume of the SMALND Group was 88.33 ± 16.79 ml, less than that of the MALND Group: 96.76 ± 26.85 ml (*P* < 0.05). The postoperative axillary mean daily drainage volume of the SMALND Group was 38.17 ± 5.55 ml, less than that of the MALND Group: 40.72 ± 7.25 ml (*P* < 0.05). The drain removal time of the SMALND Group was 7.50 ± 1.60, less than that of the MALND Group: 9.00 ± 1.80 (*P* < 0.05). The upper limb edema incidence rate of the SMALND Group was 3.12% (2/64) and had no obvious difference from the MALND Group, which was 4.83% (3/62) (*P* = 0.62). The paresthesia incidence rate of the SMALND Group was 18.75% (12/64), while that of the MALND Group was 17.7% (11/62), without an obvious difference (*P* = 0.88). For axillary lymph node dissection, the use of non-lipolytic suspension-type mastoscopy has reduced the intraoperative hemorrhage volume of patients, shortened surgery time and postoperative recovery time, saved treatment expenses for patients, and avoided complications such as hypercapnia and subcutaneous emphysema caused by traditional inflated mastoscopic surgery. Moreover, it has not increased the incidence of postoperative upper limb edema and paresthesia, supporting its safety and effectiveness.

## Introduction

Although the incidence of breast cancer increases every year, when early-stage breast cancer patients undergo standardized treatment, their five-year survival rate can be as high as over 90%. With the rapid development of modern medical and treatment technologies, life expectancy of breast cancer patients has markedly increased. It should be considered that treating breast cancer lies not only in extending the survival time of patients, but also in improving their quality of life, reducing postoperative complications, and increasing disease-free survival time^1–4^. Relevant studies on early-stage breast cancer patients have proved the safety and effectiveness of specified breast-conserving surgery (BCS)^5–10^. One of the most important objectives of BCS is to improve postoperative cosmetic effects; therefore, the periareolar incision should have advantages such as quick recovery, small scars, relatively better invisibility of surgical scars, and relatively few postoperative complications. And compared with traditional axillary lymph node dissection, Mastoscopic Axillary Lymph Node Dissection (MALND) reduces intraoperative hemorrhage volume, and shortens postoperative drain removal time and average daily drainage volume^11,12^. Furthermore, it reduces the incidence of postoperative complications, including upper limb edema and paresthesia, and reaches the objectives of minimal invasion, fast recovery, conserving function, and good appearance, making patients highly satisfied with the new dissection^13^. On the other hand, MALND could cause complications such as hypercapnia and subcutaneous emphysema: as a result, it does not suit patients of old age or with major organ disease in any part of the body.

Based on MALND, the author proposes a non-lipolytic Suspension-type Mastoscopic Axillary Lymph Node Dissection (SMALND) and analyzed and compared breast cancer patients in the SMALND and MALND Groups in terms of postoperative axillary mean daily drainage volume, axillary mean drain removal time, incidence rates of upper limb edema, and paresthesia.

## Method

### Clinical data

This study was conducted from June 2020 to April 2022 in the Department of Second Breast Surgery, Shengjing Hospital of China Medical University, and the Department of Thyroid and Breast Surgery, Zibo Central Hospital. The same group of doctors consecutively completed tracking and following up the breast cancer patients who underwent periareolar-incision breast-conserving surgery, combined with SMALND, or MALND. The screening was performed according to the inclusion and exclusion criteria for the patient groups, and a total of 126 cases met the inclusion conditions. They were randomly assigned to SMALND and MALND groups. Of them, 64 patients underwent SMALND while 62 had MALND. This study was approved by the ethics committee of Zibo central hospital [202004023]. All procedures performed in studies involving human participants were in accordance with the 1964 Helsinki Declaration and later versions. The patients signed an informed consent form for their data to be anonymously used for research purposes.

### Group inclusion criteria

1. Each patient should be informed of and consent to his or her treatment;
2. A patient should have clinical stage-I or stage-II breast cancer, without obvious skin or deep infiltration, and his or her axillary lymph node should be categorized as N1 or N0 via ultrasonic, X-ray, and breast nuclear magnetic resonance clinical examinations. However, SLNB should be performed during the procedure. The metastasis of a sentinel lymph node should be shown by intraoperative frozen sections. The TNM staging system in the NCCN Guidelines for Breast Cancer was adopted as the criteria for clinical staging^14,15^;
3. A patient should have the prescription for mastoscopic axillary lymph node dissection, and specifically: (a) have the indications for a routine axillary lymph node dissection; (b) he or she should not have a history of axillary surgery; (c) through clinical examination, ultrasound, and X-ray examination, a patient’s axillary lymph node should be less than grade N2; (d) an intumescent lymph node should not adhere to blood vessel and nerve^5^;
4. A patient should conform to the indications for breast-conserving surgery: (a) the maximum tumor diameter should be 3 cm, and after the surgery, the breast must have been able to keep proper volume and contour; (b) the tumor had not invaded nipple and areola; (c) should not have multifocal or multi-quadrant tumor lesion; (d) could undergo radiotherapy; (e) the tumor is not located at the edge of the gland and can through an areolar incision.

### Group exclusion criteria

1. A patient should not have comorbidity of any other severe chronic disease or disability, such as hypertension, diabetes, etc.;
2. A patient should not be someone who could not correctly understand and make a choice due to intelligence or mental issues;
3. A patient is not clinically above-stage-II breast cancer patient;
4. Those without indications for mastoscopic axillary lymph node dissection.

### Study methodology

The same group of doctors consecutively completed tracking and following up of the patients who underwent periareolar-incision breast-conserving surgery combined with SMALND or MALND. After screening and according to the inclusion and exclusion criteria, 126 patients met to the entry conditions. Of them, 64 underwent SMALND, and 62 had MALND. The doctors made statistics on the postoperative axillary daily drainage volume of the two patient groups, made records on their drain removal time, followed up and observed the patients on the 1st, 2nd, 7th, 14th, 30th, 60th, and 90th day after the surgery was performed. Then, by adopting the means of hospital observation, telephone counseling, or outpatient reexamination, doctors made statistics on the postoperative status of upper limb edema and paresthesia of the two patient groups.

### Surgical procedure

After a patient received general anesthesia, the surgeons had him or her take a supine position, abducted the upper limb on the patient’s side, disinfected the person with povidone iodine solution, and covered the body with a surgical drape. Then, surgeons made an incision on the areola of the side of the breast, and resected the tumor along with the surrounding gland tissue of 1 cm in the upper, lower, medial, lateral, superficial, and deep margins were sent for intraoperative frozen section. The pathologic findings indicated invasive ductal carcinoma of breast cancer, but cancer cells were not seen at the resection margins. (Aiming at the patients graded as N0 in the axillary lymph node assessment of clinical examination, ultrasonic and X-ray examination, and breast nuclear magnetic resonance examination, more methylene blue injection should be injected into the areola). Fully free the glandular skin flap in the surgical area of breast tumors, and suture the free skin flap without tumor cells around the tumor to fill the gap in the surgical area.

**Figure 1:**
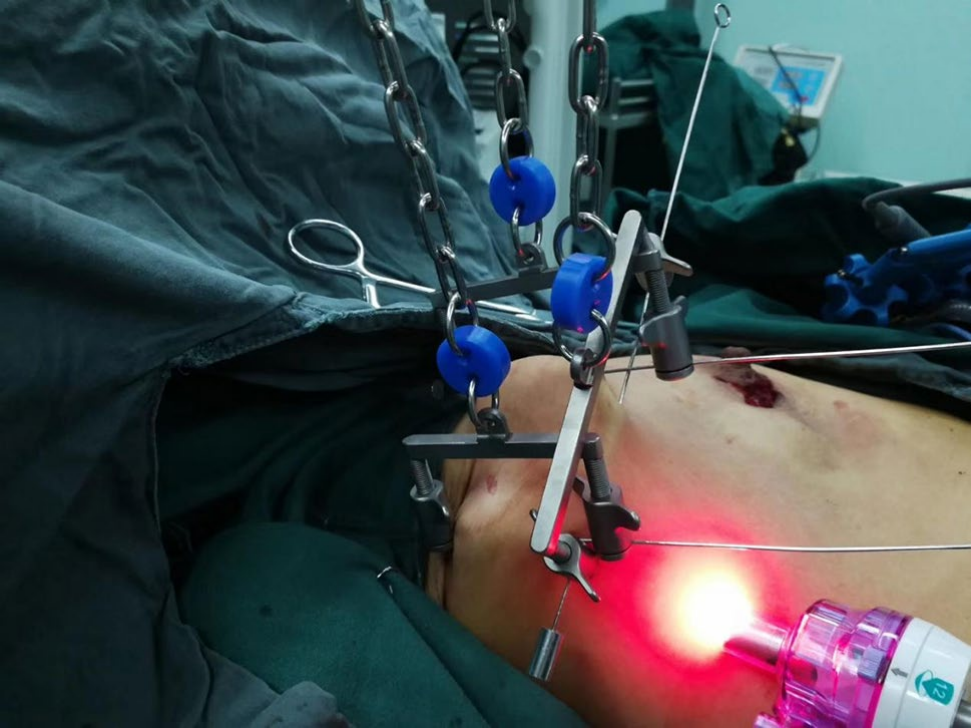
Suspended hook device free from inflation.

**Figure 2:**
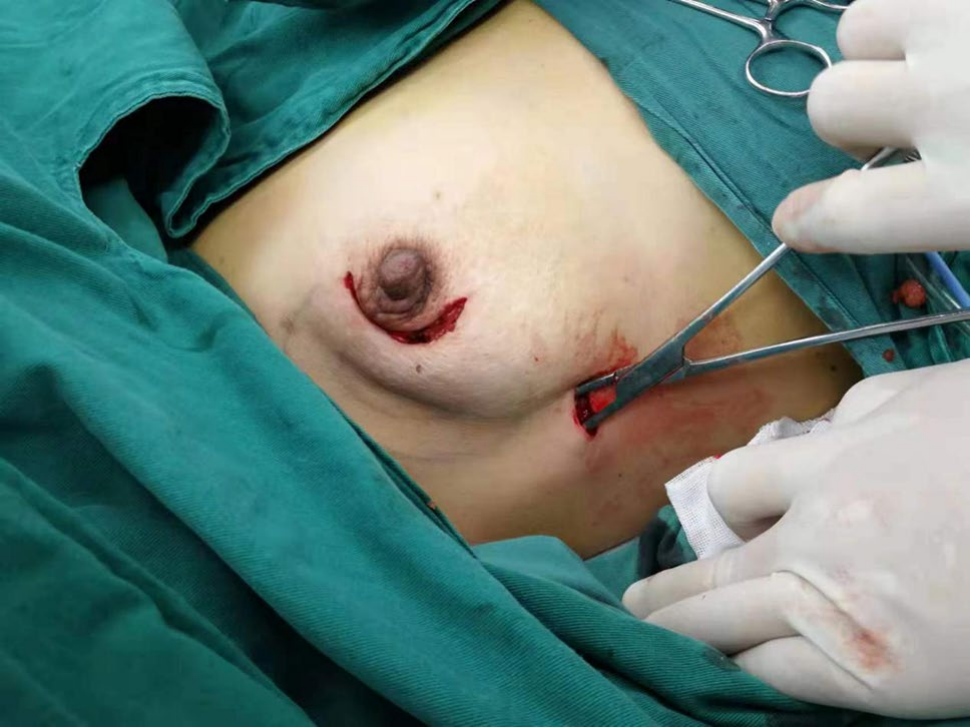
Non-lipolytic axillary blunt dissection is done for the suspension hook.

**Figure 3:**
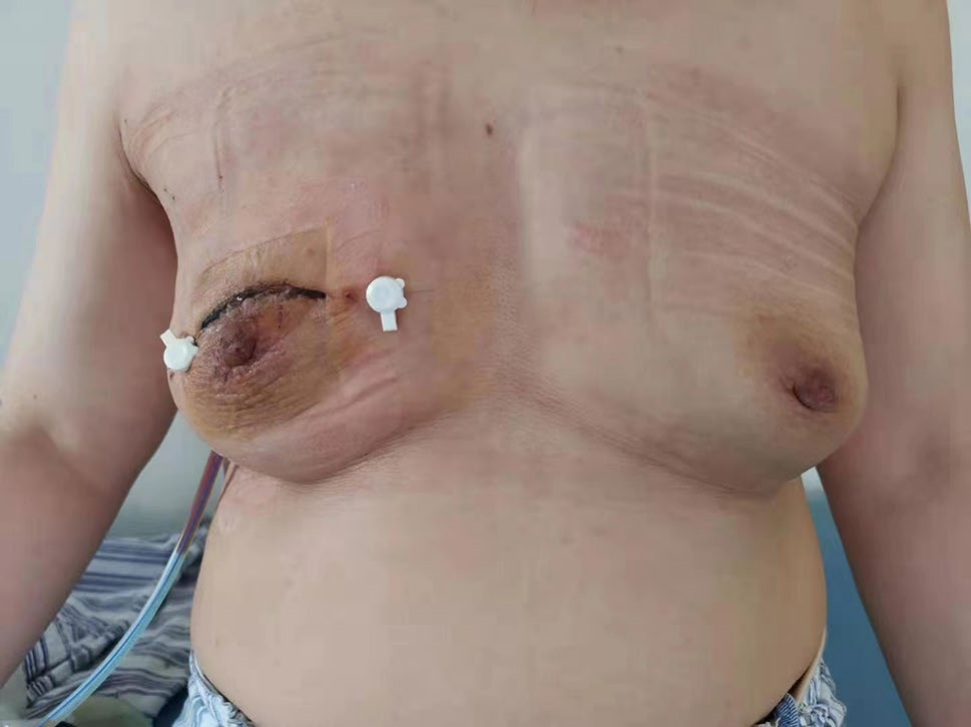
Appearance of the patient's breast after the completion of the surgery.

After suspension-type surgical devices were installed and mounted, 3 subcutaneous steel needles parallelly pierced through the skin and subcutaneous tissue at the top of the axilla to suspend the skin there (Fig. 1). A 10-mm trocar was placed from the anterior axillary line at the lower edge of the breast into the incision, and pierced along subcutaneous tissue to the axilla. The trocar was taken out and vessel forceps were used to make a blunt dissection (Fig. 2). Subsequently, an endoscope was placed through a 10-mm trocar to explore the axilla. Under the monitor of the endoscope, a 5-mm and a 10-mm trocar go into the axillary space through the breast surgery area under the monitor of the endoscope. After a successful piercing, a separating plier and a grasper were placed, respectively, into trocars. An electric hook cut and coagulate branches of blood vessels and avoided the lymph tubes observed under an endoscope. Aiming at the patients graded as N0 in the axillary lymph node assessment of clinical examination, ultrasonic and X-ray examination, breast nuclear magnetic resonance examination, etc., *sentinel lymph node biopsy* (SLNB) under mastoscopy was performed first. And intraoperative frozen sections were used to show the metastasis of a lymph node axillary dissections (ALND) of lymph node. Then dissection of axillary lymph nodes and adipose tissue were performed, then a drainage tube was indwelled where 10-mm Trocar was placed (Fig. 3). After disinfection and suture, the surgery was complete.

### MALND group

First of all, the operation of breast surgery is consistent with the SMALND group.

A tumescent fluid was prepared, with 125 ml 0.9% sodium chloride solution, 125 ml distilled water, 20 ml lidocaine, and 0.5 ml epinephrine hydrochloride. This solution was injected into the subcutaneous tissue of the axilla; a massage was applied to the area and it was let rest for approximately 20 min. Subsequently, the patient’s body position was changed, with the head raised, and the body kept a lateral tilt position. A 10-mm trocar was placed from the anterior axillary line at the lower edge of the breast into the incision and pierced along subcutaneous tissue to the axilla. After that, the fat of the area was mashed and sucted with a plastic suctor. Then, an insufflator was connected to inflate gas (CO_2_) with the internal cavity pressure maintained at 10 mmHg.

The remaining operations are the same as the SMALND group.

### Statistical analysis

All data were analyzed with the software SPSS 22.0. Normal distribution of measuring data was examined with single sample Kolmogorov–Smirnov test and expressed as mean ± standard deviation. The paired data designated as “t” were used to examine, analyze, and compare the postoperative axillary daily drainage volume and drain removal time of the two patient groups. For its part, the paired data designated as x^2^ were used to examine, analyze, and compare the incidences of upper limb edema and paresthesia in the two patient groups. Differences with *P* < 0.05 were considered statistically significant.

## Results

### Characteristics of the patients

A total of 126 patients were enrolled, of whom 64 underwent SMALND and 62 MALND. All the measuring data had normal distribution.A total of 126 patients were women.The average age in the SMALND Group was 45.44 ± 12.26 while that of the MALND Group was 45.97 ± 11.35. The difference between them was not statistically significant (*P* = 0.80). Regarding average BMI, the SMALND Group had a value of 1.65 ± 0.15 while that of the MALND Group was 1.70 ± 0.14; in the first Group, there were 40 cases of stage-T2A breast cancer and 24 cases in the stage T2B of said disease, whereas the second Group had 35 cases of T2A breast cancer and 27 cases in the stage of T2B. There were no differences between the two groups in the clinical and pathological characteristics of the patients and cancers (all *P* > 0.05) (Table 1).

**Table 1:** Characteristics of the patients.

Clinical data	SMALND (n = 64)	MALND (n = 62)	*P*
Age (years)	45.44 ± 12.26	45.97 ± 11.35	0.80
BMI (kg/m^2^)	23.67 ± 2.92	23.62 ± 2.78	0.60
Stage			
T2A	40	35	0.58
T2B	24	27	
Subtype			
Luminal A	0	0	1.0
Luminal B (HER-)	32	27	0.47
Luminal B (HER+)	6	5	0.79
HER2+	11	8	0.62
Triple-negative	15	22	0.17
ER-positive	38	32	0.38
PR-positive	35	27	0.21
HER2-positive	17	13	0.46

*SMALND* suspended axillary lymph node dissection, *MALND* mastoscopic axillary lymph node dissection, *ER* estrogen receptors, *PR* progesterone receptor.

### Surgical characteristics

Intraoperative blood loss, axillary processing time and daily drainage, and average drain removal time in SMALND group were lower than in MALND group. There were no significant differences in axillary lymph nodes between the two groups (*P* = 0.14) (Table 2).

**Table 2:** Surgical characteristics.

Clinical data	SMALND	MALND	*P*
Intraoperative blood loss (ml)	88.33 ± 16.79	96.76 ± 26.85	0.01
Axilla processing time (min)	43.38 ± 6.27	45.72 ± 4.25	0.02
Average daily axillary drainage (ml)	38.17 ± 5.55	40.771 ± 7.02	0.02
Drain removal time (days)	7.5 ± 1.6	9.0 ± 1.8	0.02
Number of lymph node dissection	15.98 ± 4.11	15.00 ± 3.28	0.14
Treatment of lymph nodes n (%)			
SLNB	10	8	0.72
ALND	54	54	

### Postoperative complications

#### Upper limb edema incidence rate

7th day: The patient 's general elastic bandage oppresses the axillary area for 10 days after surgery. At this time, upper limb edema cannot be rule out as the pressure bandage oppresses the axillary too tightly and may affect lymphatic reflux and drainage. So the results may have certain errors. The rate was 3.12% (2/64) in the SMALND Group, and 4.83% in the MALND Group, without differences between the groups (*P* = 0.62). On this day, 2 cases occurred among the patients from the SMALND Group. Of them, 1 had mild edema in the upper limb without affecting functions; the swelling scope was limited to the upper arm, where pitting edema appeared under pressure, but resolved by raising the arm. In the other case, swelling occurred in the upper arm, with functions affected to a certain extent; the swelling extended to the forearm, with hard and solid surface, and without pitting edema. In the MALND Group, 3 cases of edema occured among the patients, of whom, in 2 cases, mild edema in the upper limb, without affecting functions, and was limited to the upper arm, where pitting edema appeared under pressure, but resolved with the arm raised; in the other case, swelling occurred in the upper arm, with functions affected to a certain extent, and the swelling extended to the forearm, with hard and solid surface and without pitting edema.

30th day: the rate was 1.56% (1/64) in the SMALND Group, and 3.23% (2/62) in the MALND Group, without differences between the groups (*P* = 0.54). On this day, 1 case occurred in the SMALND Group and had the same clinical manifestations as the patient with mild edema from day 7. In the MALND Group, there were 2 cases of upper limb edema. In both cases, mild edema occurred in the upper limb, without affecting functions; the swelling was limited to the upper arm, where pitting edema appeared under pressure, and resolved when the arm was raised.

90th day: the rate was 0% (0/64) in the SMALND Group, and 0% in the MALND Group, without differences between the groups (*P* = 1.00).

We followed up the patients with upper limb edema for only 3 months, so this conclusion may be biased.

#### Paresthesia incidence rate

7th day: the rate was 18.75% (12/64) in the SMALND Group and 17.74% (11/62) in the MALND Group, without differences between the groups (*P* = 0.88). In the SMALND Group, 12 cases of paresthesia occurred, of whom all had Grade-I to Grad-II pain, 4 had hypoesthesia or anesthesia, and 2 had soreness and swelling, or a sense of heaviness. In the MALND Group, 11 cases of paresthesia occurred, of whom 8 had Grade-I to Grad-II pain, 5 had hypoesthesia or anesthesia, and 7 had soreness and swelling, or a sense of heaviness.

30th day: the rate of the patients was 6.25% (4/64) in the SMALND Group and 6.45% (4/62) in the MALND Group, without differences between the groups (*P* = 0.96). On this day, 4 cases occurred in the SMALND Group, of whom 4 had Grade-I to Grad-II pain, and 3 had hypoesthesia or anesthesia. In the MALND Group, there were 4 cases of paresthesia, of whom 4 had Grade-I to Grad-II pain, 2 had hypoesthesia or anesthesia, and 1 case had soreness and swelling, or a sense of heaviness.

90th day: the rate was 4.69% (3/64) in the SMALND Group and 3.23% (2/62) in the MALND Group, without differences between the groups (*P* = 0.43). In the SMALND Group, there were 3 cases of paresthesia, of whom 3 had Grade-I to Grad-II pain, and 3 had hypoesthesia or anesthesia. In the MALND Group, there were 2 cases of paresthesia, of whom 1 had Grade-I to Grad-II pain, and 2 cases had hypoesthesia or anesthesia. The pain scale corresponded to the NCCN Guidelines^14^.

## Discussion

In this breast cancer study, two approaches for periareolar-incision breast-conserving surgery were compared: first, a combination with traditional MALND, and, second, a combination with SMALND. The second case had little postoperative drainage volume, short drain removal time with quicker recovery, and satisfying breast conservation. There were no significant differences in postoperative complications between the two groups. The surgery helped treat this disease, while it also conserved the patient’s breasts. As a result, it helps improve the quality of life of the patient.

The postoperative body image of a breast cancer patient severely affects his or her wellbeing^16^. Some sensitive patient requirements for the surgery are almost invisible surgical scars and the least obvious deformity of breasts. Therefore, in most cases, patients and doctors decide on surgical incisions by BCS not only to resect a tumor, but also to benefit from suitable cosmetic effects. To make surgical scars invisible or less evident, the incision is usually applied close to axillary wrinkles, the lower breast wrinkles formed by the natural mastoptosis of breast, or in the periareolar area, because an incision at these positions can offer the best cosmetic effects. The periareolar technique can resect any quadrant of the tumor, including benign breast tumors, or even the BCS surgery far from NAC. Periareolar incision the advantages of quick recovery, small scars, relatively better invisibility of surgical scars (good cosmetic effects), and relatively few postoperative complications.

When the tumor is in a non-central area, the tissue behind the areola can be kept. With the present approach, surgical incisions rarely exceed 1/4 of the perimeter of the areola to avoid damage to the blood vessels of the flap as much as possible. Therefore, complications of NAC ischemia rarely occur. The size of the areola directly affects the length of the incision; thus, to ensure surgical operation and vision, the scope of the incision exceeds a quadrant. However, to ensure the blood supply of NAC is not affected, the size of the periareolar incision should not exceed half of the total perimeter of areola.

When the tumor is far from the areola and located at the periphery of a gland, it is important to resect the tumor if the outer gland is slightly pushed from outside of the tumor toward NAC. If, before the surgery, it is unclear whether the tumor can be touched, the tumor should be accurately located from the body surface via ultrasound after the patient’s surgical position has been fixed. This is crucial to accurately resect the tumor and cut as less normal glands as possible, which is needed not to change the breast appearance. Under the premise that the superficial and basal negative resection margin is ensured, the superficial and deep adipose tissue of the tumor is not completely resected because a small amount of those kinds of tissues are not related to local recurrence. Nevertheless, their preservation is essential to keep the normal contour of the breast and obtain satisfactory cosmetic effects^17^.

Endoscope-aided techniques for the application of breast-conserving surgery have mainly resolved the problem of notorious auxiliary incision scars, as seen after traditional breast-conserving surgery combined with axillary lymph node dissection. Mastoscopic axillary lymph node dissection has become a widely used surgical option for axillary lymph node dissection in breast cancer patients. Literature shows that, after these patients undergo mastoscopic axillary lymph node dissection, their postoperative recovery time is short, and the incidence of complications, including upper limb edema and paresthesia, is evidently lower than that of traditional axillary lymph node dissection^18–20^.

Through mastoscopic axillary lymph node dissection, axillary nerves, blood vessels, and lymph tubes can be magnified by 8–10 times via endoscope, which allows seeing lymph nodes, axillary veins, thoracodorsal nerves and blood vessels, intercostobrachial nerves, and lymph tubes. During the procedure, the dissection also mostly retains intercostobrachial nerve, avoids damage to blood vessels, nerves, and lymph tubes, evidently reduces intraoperative hemorrhage and postoperative hemorrhage, shortens the postoperative time of indwelling drainage tube, and lowers the risks of the incidence of postoperative upper limb edema and paresthesia. These outcomes improve patients’ postoperative survival and quality of life.

During the procedure of mastoscopic axillary lymph node dissection, the establishment of axillary space has become one of the key aspects; a narrow and small axillary space is usually established by inflation. Axillary space established through traditional inflation is relatively unstable because the surgical vision can be reduced due to air leakage; moreover, the mist produced by the hemorrhage of minute blood vessels and the hemostasis with an electric hook in the small space results in the blur of the lens, which severely affects the operation and prolongs surgical time. Furthermore, if CO_2_ gas is inflated beyond appropriate, it can cause harmful effects on the physiological functions of patients’ visceral organs and thermal damage to their organs, especially for elderly patients complicated with underlying diseases. Over-inflation of CO2 can increase the risks of subcutaneous emphysema, gas embolism, hypercapnia, venous reflux block, shoulder pain, nausea, and vomiting^21–23^.

In order to resolve the deficiencies of traditional inflated surgery and their complications, the author has presented the surgical method of non-lipolytic SMALND. In this procedure, mechanical devices are used to raise and pull axillary surface skin to replace the space demanded by mastoscopic surgery. After a 10-mm Trocar incision, vessel forceps were used to make a blunt dissection and create a lacuna that could avoid the allergy caused by solute process^24^. The omission of injection of the solution into blood vessels reduces the harm to axillary soft tissue, blood vessels, and lymphatics caused by lipolysis.

The operation time of SMALD was shorter than that of MALND. Patients recover quickly after the surgery, and drain removal time and hospital discharge time were noticeably better than performing MALND after solute process.

In this study, in terms of postoperative complications, there were no significant differences between the 64 patients undergoing SMALND, and the 62 patients undergoing traditional inflated mastoscopic surgery. SMALND could be used to completely replace traditional inflated mastoscopy because it can save economical expenses for patients, avoid complications such as subcutaneous emphysema, gas embolism, and hypercapnia, and also reduce surgical time and intraoperative hemorrhage volume. As a result, it enhances the recovery of patients and shortens hospitalization time.

The medical device for the suspension-type procedure does not occupy any axillary space; the only requirement is to suspend the axilla from the skin surface through disinfected subcutaneous steel needles. The equipment can be installed and operated simply, and mounted reliably. On the other hand, there is almost no scar after the surgery, and there the inflated devices do not add clinical risks. This way, safe, stable and sufficient axillary operation space can be created for mastoscopic surgery. After the axillary space is formed, the endoscope can offer a clearer and wider axillary view to overcome the difficulties of narrow and small spaces typical of mastoscopic surgery. The classic problems found in traditional inflated surgery such as instability in the surgical space for using inflated devices, limitation to surgical operation, and complications such as subcutaneous emphysema and hypercapnia caused by CO_2_, can be overcome by implementing this suspension-type procedure.

The major deficiency of the suspension-type mastoscopic surgery is that it creates space after the pulling and raising force of suspension bars and steel-needle tongs, whose angles need to be adjusted to reach the equilibrium of space. However, this problem does not affect conducting the operation and its effects. It is suggested to use at least 3 steel-needle tongs to fix the axilla; this way, the axilla can be fully exposed. The axillary space is made through a blunt dissection and it avoids the allergy caused by a solute process. The omission of injection of the solution into blood vessels reduced the damage to axillary soft tissues, blood vessels, and lymph tubes caused by violent lipolysis in solute processes, and markedly shortened patients’ hospitalization time and drain removal time, promoting a quick recovery.

At the same time, this study has some limitations. The number of cases enrolled in this study was small, and the observation time of postoperative complications was relatively short. Whether SMALND can replace MALND needs further big data clinical research.

In conclusion, for patients in the early stages of breast cancer, periareolar-incision breast-conserving surgery combined with non-lipolytic SMALND is a safe and feasible surgical method, and a major breakthrough in mastoscopic surgery, with substantial clinical value.

## Data Availability

The datasets used and/or analyszed during the current study are available from the corresponding author on reasonable request.

## Abbreviations

SMALND: Suspension-type Mastoscopic Axillary Lymph Node Dissection
BCS: Breast-conserving surgery
ALND: Axillary lymph node dissection
MALND: Mastoscopic Axillary Lymph Node Dissection
SLNB: Sentinel Lymph Node Biopsy
NAC: Nipple-Areolar Complex
